# Special Issue “Molecular Mechanisms of Neurological and Psychiatric Disease: A Decade of Progress”

**DOI:** 10.3390/ijms262110320

**Published:** 2025-10-23

**Authors:** Giuseppina Martella, Martina Montanari

**Affiliations:** 1 Department of Psychology and Health Sciences, Faculty of Humanities, Education and Sports, Pegaso University, 80145 Naples, Italy; 2 Laboratory of Neurophysiology and Plasticity, IRCCS Fondazione Santa Lucia, 00179 Rome, Italy; m.montanari@hsantalucia.it; 3 Department of Systems Medicine, University of Rome Tor Vergata, Via Montpellier 1, 00133 Rome, Italy

## 1. Introduction

This Special Issue, “Molecular Mechanisms of Neurological and Psychiatric Disease: A Decade of Progress”, explores the dissolving boundaries between neurological and psychiatric disorders at the molecular level.

Psychopharmacology’s precise mechanisms of action have revolutionised treatment, but a critical knowledge gap still needs to be addressed. This Special Issue includes five original research articles that aim to elucidate the underlying molecular connections between these interconnected pathologies.

For decades, psychological and behavioural therapies have been the foundations of mental health treatment. However, the advent of modern psychopharmacology has profoundly reshaped the therapeutic landscape, integrating medications to address a wide array of psychiatric conditions. Despite these revolutionary advances, a critical challenge remains: the precise mechanisms of action of many pharmacological treatments remain largely unknown. This persistent knowledge gap compels us to explore further.

It has become increasingly evident that psychiatric and neurological disorders are profoundly interconnected, blurring conventional diagnostic boundaries and demanding more integrative, multidisciplinary approaches to patient care [1]. While traditionally viewed as distinct disciplines, our deepening understanding of brain function has revealed a more complex picture. Neurological diseases, typically defined by disruptions in neuronal circuitry, are frequently accompanied by psychiatric symptoms such as depression, anxiety, and cognitive impairment [1,2]. Addressing these psychiatric manifestations is not merely a matter of accurate diagnosis and treatment; it is essential for mitigating the broader psychosocial impact on patients, their families, and their caregivers [3]. To deliver truly effective and compassionate care, a comprehensive understanding of these complex interactions is required [4].

However, it is a striking paradox that despite their clear clinical overlap and significant impact on quality of life, these comorbid conditions are often underdiagnosed or neglected, possibly because of shared neurochemical pathways or a general decline in functional capacity [5,6,7].

This challenge extends beyond diagnosis. Current pharmacological treatments have notable limitations, including adverse side effects, drug interactions, and suboptimal efficacy. This underscores the urgent need for a deeper understanding of the underlying biology. In recent decades, researchers’ tireless efforts have led to fundamental breakthroughs in the pathophysiology of these disorders, clarifying disease progression and identifying crucial new molecular targets for therapeutic intervention [6,7,8,9].

Looking ahead, it is clear that the key to developing more effective and targeted therapies lies in elucidating the molecular mechanisms underlying these interconnected pathologies. We can move beyond a purely symptomatic approach by embracing a perspective recognising the synergy between neurology and psychiatry. This shift paves the way for a new era of precision medicine, a crucial step toward transforming mental healthcare and tangibly improving the lives of countless individuals.

## 2. Research

Based on the intricate connections between neurology and psychiatry and the pressing need to unravel their molecular mechanisms, this Special Issue of *IJMS* includes five original research articles that significantly contribute to the field. Each study is representative of the current research landscape, focusing on specific pathologies and providing valuable insights into topics ranging from genetics and the discovery of novel biomarkers to exploring innovative therapeutic strategies.

In a groundbreaking study, Kuo and coworkers explored the neuroprotective potential of a novel non-pharmacological approach, thermal cycling–hyperthermia (TC-HT). In a cellular model of Parkinson’s disease (PD), TC-HT effectively mitigated rotenone-induced mitochondrial apoptosis and oxidative stress in SH-SY5Y cells. The authors provide a detailed mechanistic explanation, showing that this treatment downregulates pathological markers such as α-synuclein and phosphorylated tau by activating key heat-response pathways involving SIRT1, Hsp70, and Akt/GSK-3β signalling. These findings suggest a promising drug-free strategy that could complement existing therapies and potentially offer a new avenue for PD management (**Contribution 1**).

Agúndez’s group delved into the genetic landscape of essential tremor (ET), a movement disorder with complex pathophysiology. They investigated the genetic association between LAG3/CD4 single-nucleotide variants (SNVs) and ET risk in the Spanish population. Although their analysis did not find a strong association, it revealed a notable marginal protective effect of the LAG3 rs870849C variant in male patients. These results are particularly relevant as they contribute to the ongoing scientific debate regarding the genetic overlap between ET and Parkinson’s disease. Their work highlights the critical role of immune-related genes in the pathophysiology of tremors and calls for further research into specific genetic predispositions in different patient subgroups (**Contribution 2**).

In a significant contribution to neurodegenerative diseases, Lin and collaborators focused on spinocerebellar ataxia type 3 (SCA3), a progressive disorder characterised by the aggregation of mutant proteins. They identified and characterised a series of synthetic indole and coumarin derivatives that act as dual-function chemical chaperones and autophagy inducers. The authors demonstrated that these compounds promote the degradation of mutant ATXN3 aggregates by enhancing LC3-mediated autophagic flux. They further revealed a direct physical interaction between these compounds and mutant ATXN3 and LC3, introducing a new class called autophagosome-tethering compounds (ATTECs). This study offers a highly innovative therapeutic strategy based on a precise molecular mechanism (**Contribution 3**).

Fila and coworkers provide valuable insights into the search for objective biomarkers for migraine, a notoriously subjective condition that is difficult to diagnose and manage. Their study examined the relationship between urinary 5-hydroxyindoleacetic acid (5-HIAA) levels and migraine characteristics during the interictal phase of episodic migraine. The results revealed a negative correlation between 5-HIAA levels and the frequency of migraine attacks. This finding suggests that a marker of tryptophan metabolism may serve as a potential and much-needed biomarker for both the diagnosis and monitoring of migraine, paving the way for more precise and objective clinical assessments that could improve patient care (**Contribution 4**).

Finally, Park and collaborators addressed a critical challenge in migraine research: understanding the transition from episodic to chronic migraine. Their study utilised distinct mouse models of episodic and chronic migraine induced by nitroglycerin to compare neuropathological features. Their comprehensive analysis highlighted the molecular and cellular changes associated with migraine chronification. These findings offer a deeper understanding of the mechanisms underlying disease progression, providing a solid foundation for developing targeted interventions to prevent this debilitating transition and improve the quality of life of millions of individuals (**Contribution 5**).

## 3. Review

In addition to the original research articles, this Special Issue presents two comprehensive reviews offering critical and forward-thinking perspectives on emerging molecular strategies for preventing and treating neurological and psychiatric disorders. These contributions reflect the evolving landscape of translational research, wherein molecular insights are increasingly integrated with systemic, lifestyle, and nutritional approaches to improve patient outcomes [10].

The first review by Montanari, Mercuri, and Martella (**Contribution 6**) addressed the growing challenge of physical and cognitive frailty, particularly within the context of Parkinson’s disease (PD). These conditions, which are increasingly prevalent in ageing populations, share overlapping molecular mechanisms, such as oxidative stress, mitochondrial dysfunction, and neuroinflammation. The authors meticulously explored the therapeutic potential of nutraceuticals (naturally occurring bioactive compounds) as adjunctive interventions capable of modulating these pathways. This review proposes a holistic framework integrating nutraceuticals with physical activity, conventional pharmacological treatments, and lifestyle modifications by synthesising a decade of evidence. This approach is not merely about neuroprotection; it aims to improve muscle function, enhance cognitive resilience, and ultimately elevate the overall quality of life in older adults. This review exemplifies the commitment of this Special Issue to multidimensional therapeutic innovation grounded in a deep molecular understanding.

Baj and coworkers conducted the second review (**Contribution 7**) on the nascent but rapidly growing field of metallomics in psychiatry, with a particular focus on major depressive disorder (MDD). The authors systematically evaluated the role of 15 trace elements, including zinc, magnesium, selenium, copper, and iron, in the onset and progression of depressive symptoms. Their rigorous analysis revealed that deficiencies and excesses of specific elements can influence neurochemical signalling, oxidative balance, and immune responses, thereby contributing to the multifactorial aetiology of MDD. Notably, this review highlights the potential of trace element profiling as a diagnostic and prognostic tool, informing personalised treatment strategies and identifying individuals at risk. This contribution reinforces the emphasis on molecular biomarkers and the integration of nuanced biochemical data into psychiatric care, promising a more precise and tailored approach to mental health.

Together, these reviews expand the thematic scope of the Special Issue by bridging molecular mechanisms with systemic and environmental factors. They powerfully underscore the importance of interdisciplinary approaches in addressing the immense complexity of neurological and psychiatric diseases, offering a vision for the future of medicine that is both comprehensive and compassionate [10].

Further reinforcing the central aim of this Special Issue, an additional noteworthy contribution offers a compelling link between two seemingly distinct areas of neuropathology: ischemic injury and Alzheimer’s disease.

In their insightful article, Pluta and Czuczwar (**Contribution 8**) investigate the molecular drivers of neurodegeneration following post-ischemic brain injury. Their work draws a powerful parallel with Alzheimer’s disease (AD) by exploring the role of trans- and cis-phosphorylated tau protein in the formation of neurofibrillary tangles. The authors provide compelling evidence that a brain ischemic event may initiate a cascade of molecular events strikingly similar to those seen in AD, including the accumulation of amyloid plaques and tau hyperphosphorylation. They focus on the hippocampus, a brain region critically involved in memory processing, demonstrating how this damage can be a molecular trigger for long-term neurodegeneration. A key finding of their study is the pathological significance of the Thr231 motif in the tau protein, which exists in two distinct conformations—trans and cis. The presence of these conformations acts as a crucial precursor to tauopathy. This mechanistic insight suggests that ischemic injury may not only cause immediate damage but also serve as a driver of AD-like neurodegeneration, thus opening new perspectives on the intricate overlap between vascular and neurodegenerative processes.

This study aligns perfectly with the overarching goals of the Special Issue. Meticulously detailing the shared molecular pathways across these distinct neurological conditions contributes to our fundamental understanding of disease. It identifies potential early biomarkers and therapeutic targets for tau-related neurodegeneration, highlighting the importance of a comprehensive approach to brain health.

## 4. Conclusions

The articles featured in this Special Issue, Molecular Mechanisms of Neurological and Psychiatric Disease: A Decade of Progress, collectively contribute to a deeper and more nuanced understanding of the molecular foundations of complex brain disorders. Through original research and comprehensive reviews, this collection highlights the diverse mechanisms underlying neurological and psychiatric conditions, ranging from mitochondrial dysfunction and oxidative stress to autophagy, trace element imbalance, and protein misfolding.

Crucially, these studies support the initial aim of this Special Issue: to explore how shared molecular pathways can inform the development of more effective, personalised, and integrative therapeutic strategies. For example, recent research underscores the importance of the gut–brain axis, demonstrating that gut microbiota can influence neuroinflammation and alter neurotransmitter levels, impacting mood disorders and neurodegenerative conditions [11]. This perspective is further supported by studies showing that lifestyle and dietary interventions can modulate these pathways, enhancing neuroprotection and complementing pharmacological treatment [12].

Similarly, mitochondrial dysfunction is gaining recognition as a key player in neurological and psychiatric diseases. Accumulating evidence reveals that impaired mitochondrial function contributes to neuronal damage in diseases such as Parkinson’s and Alzheimer’s, as well as to the pathophysiology of major depressive disorder and bipolar disorder [13,14]. These insights suggest that targeting mitochondrial health may be a promising cross-disciplinary therapeutic strategy.

Furthering this line of inquiry, research on proteostasis, which regulates protein folding and degradation, reveals a fundamental shared vulnerability. Dysregulation of protein homeostasis, which leads to the accumulation of misfolded proteins, is a hallmark of neurodegenerative diseases. However, it is also increasingly linked to psychiatric conditions, such as schizophrenia and bipolar disorder [15]. These findings underscore a typical molecular architecture that transcends the traditional diagnostic categories.

The contributions in this Special Issue reflect a growing consensus in the field that the boundaries between neurological and psychiatric disorders are increasingly blurred at the molecular level. By identifying shared mechanisms and novel biomarkers, these studies pave the way for precision medicine approaches to improve the diagnosis, treatment, and long-term outcomes of a broad spectrum of brain diseases.

## References

[1] Martinez-Ramirez S., Pany M. (2022). Neuropsychiatric Disorders in Parkinson’s Disease. Neurol. Sci. Ther..

[2] Chun H.Y., Ford A., Kutlubaev M.A., Almeida O.P., Mead G.E. (2022). Depression, Anxiety, and Suicide After Stroke: A Narrative Review of the Best Available Evidence. Stroke.

[3] Lyketsos C.G., Kozauer N., Rabins P.V. (2007). Psychiatric manifestations of neurologic disease: Where are we headed?. Dialogues Clin Neurosci..

[4] Maristany A.J., Sa B.C., Murray C., Subramaniam A.B., Oldak S.E. (2024). Psychiatric Manifestations of Neurological Diseases: A Narrative Review. Cureus.

[5] Richerson G.B., Buchanan G.F. (2011). The serotonin axis: Shared mechanisms in seizures, depression, and SUDEP. Epilepsia.

[6] Batulan Z., Bhimla A., Higginbotham E.J., National Academies of Sciences, Engineering, and Medicine, Health and Medicine Division, Board on Population Health and Public Health Practice, Committee on a Framework for the Consideration of Chronic Debilitating Conditions in Women (2024). Advancing Research on Chronic Conditions in Women.

[7] Götze H., Taubenheim S., Dietz A., Lordick F., Mehnert A. (2018). Comorbid conditions and health-related quality of life in long-term cancer survivors-associations with demographic and medical characteristics. J Cancer Surviv..

[8] Stancheva M., Dimcheva S., Petkova L. (2022). Advances in Genetic and Molecular Understanding of Alzheimer’s Disease. Int. J. Mol. Sci..

[9] Gribkoff V.K., Kaczmarek L.K. (2023). The Difficult Path to the Discovery of Novel Treatments in Psychiatric Disorders. Adv. Neurobiol..

[10] Fernandes J.B., Baixinho C., Outeiro T.F., Godinho C. (2024). Editorial: One health care in psychiatric and neurological diseases. Front. Psychiatry.

[11] Cryan J.F., O’Riordan K.J., Cowan C.S.M., Sandhu K.V., Bastiaanssen T.F., Boehme M., Codagnone M.G., Cussotto S., Fulling C., Golubeva A.V. (2019). The Microbiota-Gut-Brain Axis. Physiol. Rev..

[12] Lovelace M.D., Varney B., Sundaram G., Lennon M.J., Lim C.K., Jacobs K., Guillemin G.J., Brew B.J. (2017). Recent evidence for an ex-panded role of the kynurenine pathway of tryptophan metabolism in neurological diseases. Neuropharmacology.

[13] Picard M., McEwen B.S. (2018). Psychological Stress and Mitochondrial Energetics. Neuroscience.

[14] Ashleigh T., Swerdlow R.H., Beal M.F. (2023). The role of mitochondrial dysfunction in Alzheimer’s disease pathogenesis. Alzheimers Dement..

[15] Laguesse S., Ron D. (2020). Protein Translation and Psychiatric Disorders. Neuroscientist.

